# Recent Integrations of Latent Variable Network Modeling With Psychometric Models

**DOI:** 10.3389/fpsyg.2021.773289

**Published:** 2021-12-09

**Authors:** Selena Wang

**Affiliations:** Department of Psychology, The Ohio State University, Columbus, OH, United States

**Keywords:** network analysis, psychometrics, network psychometrics, latent space models, item response models, latent space item response models, joint latent space models

## Abstract

The combination of network modeling and psychometric models has opened up exciting directions of research. However, there has been confusion surrounding differences among network models, graphic models, latent variable models and their applications in psychology. In this paper, I attempt to remedy this gap by briefly introducing latent variable network models and their recent integrations with psychometric models to psychometricians and applied psychologists. Following this introduction, I summarize developments under *network psychometrics* and show how graphical models under this framework can be distinguished from other network models. Every model is introduced using unified notations, and all methods are accompanied by available R packages inducive to further independent learning.

## 1. Introduction

Networks represent relationships or edges among a group of entities (we will call nodes), which, depending on the context, can be individuals, cells, countries, railway stations, and ecological species. Systems resembling a network are abundant with examples such as the World Wide Web, power grid systems, cell networks, economical networks, and social networks. The most commonly studied networks in behavioral science perhaps are social networks. Social networks can arise in various contexts including follower-followee relationships in online platforms, coauthorship among scientists, and friendships formed at schools.

Statistical modeling of networks has emerged as a major topic of interest, with a growing number of books and survey papers across disciplines (e.g., Wasserman and Faust, 1994; Newman, 2003; Goldenberg et al., 2010; Snijders, 2011; Vivar and Banks, 2012; Matias and Robin, 2014; Sweet, 2016; Desmarais and Cranmer, 2017; Kim et al., 2018; Smith et al., 2019; Zhang et al., 2020; Sosa and Buitrago, 2021). Existing surveys cover a broad range of topics including processes occurring on networks, exponential random graph models (ERGMs), stochastic actor oriented models (SAOMs), and latent variable network models, e.g., latent space models (LSM) and stochastic blockmodels (SBM).

A latent variable is not directly observed, but inferred from observed variables. A latent variable can be used to reduce the complexity of information by providing a parsimonious description of a multitude of noisy and often high-dimensional observations. This benefit of latent variables has been seen in a variety of settings, including factor analysis, item response theory (Spearman, 1904; Harman, 1976; van der Linden and Hambleton, 1997), and more recently, in network modeling (Snijders, 1996; Hoff et al., 2002). The key benefit of latent variables in network modeling is to capture various forms of dependencies or associations between a pair of persons or a group of persons. Conditional on latent variables, some form of independence can be assumed in the error term (Henry, 2014), which largely simplifies the model. In educational and psychological studies, latent variables are also used to model abstract concepts such as depression, extraversion, and intelligence. I refer readers to Bollen (2002) for a further discussion on the use of latent variables in psychology.

With the rising popularity of network modeling, there has been rapid growth in research works that incorporate latent variable network models with psychometric models. Given the rather new status of this type of research, to the best of my knowledge, there has not been any attempt to document the progress in the integrations of latent variable network models with psychometric models such as item response models, structural equation models, and factor analysis. Therefore, in this paper, I outline key developments bridging network latent variable models with psychometric models, summarize their connections with network models as well as psychometric models and point out directions for future research.

The paper is organized as follows. I first introduce network modeling using discrete and continuous latent variables, i.e., stochastic blockmodels and latent space models. I divide latent space models into two categories following models' latent effects, whether the latent effect is based on Euclidean distances or vector products. I then describe integrative frameworks motivated by latent variable network models and psychometric models. In section 4, I summarize model developments under the popular *network psychometrics* framework and provide key distinctions of this framework from the current topic. In section 5, I present a diagram summarizing relationships of all models and point out current gaps in research and possible future directions.

## 2. Latent Variable Network Modeling

In this section, I first describe network modeling using discrete latent variables, namely stochastic blockmodels. Then, I introduce two categories of network models with continuous latent variables: (i) distance models that are built on Euclidean distances between two nodes, and (ii) vector models that are built on vector products between two nodes. The reasoning behind this categorization is two-fold. First, these two categories represent two distinct ways of modeling information in a network and result in two distinct ways of interpreting latent spaces. Second, there are many models under each of these two categories that would be difficult to keep track of if this categorization was not made.

The purpose of discussing latent variable network modeling is two-fold. First, despite many efforts surveying network models, a detailed introduction of latent variable network models to behavioral scientists is still lacking. By providing a comprehensive survey of latent variable network models using unified notations and terminologies, I hope to provide an introductory tool for psychologists from substantive as well as methodological backgrounds to delve deeper into this topic. Second, in the next section, it can be seen that among many available options for latent variable network models, only one or two are chosen by psychologists. Therefore, I hope to encourage a more diverse integration of network models with psychometric models by providing a comprehensive discussion of latent variable network models.

### 2.1. Stochastic Blockmodels

In this section, I summarize the *posteriori* blockmodeling of networks introduced by Snijders and Nowicki (1997) and Nowicki and Snijders (2001). In the stochastic blockmodels, the probability of a connection between two nodes depends on their block (group) memberships. Suppose there is a network written as an adjacency matrix:


(1) $$\begin{array}{l} X=\left[\begin{array}{cccc} x_{1,1} & x_{1,2} & \ldots & x_{1,N}\\ \vdots & \vdots & \ddots & \vdots \\ x_{N,1} & x_{N,2} & \ldots & x_{N,N} \end{array}\right], \end{array}$$

where *x*_*a,b*_ represents the presence of an edge (binary network) or the degree of association (weighted network) between nodes *a* and *b*, *a, b* = 1, …, *N* and *b* ≠ *a*^1^, and *N* is the total number of nodes. Let us define the discrete latent variable *u*_*a*_ as the latent block membership for node *a*, *u*_*a*_ ∈ 1, 2, …, *K*, where *K* is the total number of blocks or groups. Conditional on the latent variable, the probability of two nodes forming an edge is independent of all the other edges in the network:


(2) $$\begin{array}{l} P\left(X|U\right)=\overset{N}{\underset{a=1}{\prod }}\overset{N}{\underset{b=1,b\neq a}{\prod }}p\left(x_{a,b}|u_{a},u_{b}\right),\;p\left(x_{a,b}|u_{a},u_{b}\right)=m_{u_{a},u_{b}}, \end{array}$$

where *m*_*u*_*a*_, *u*_*b*__ is the probability of a connection between the corresponding blocks. SBMs can be used to identify equivalent nodes in a network based on their relationships with other nodes (structural equivalence) as members of the same group share the same patterns of relationships. Stochastic blockmodels can be fitted using the CIDnetworks package with MCMC algorithms (Adhikari et al., 2015) and the blockmodels package using variational inference (Leger, 2015), among others (e.g., Brusco et al., 2020; Chiquet et al., 2021).

However, the assumption that the probability of shared connections between nodes is dependent on their membership to a single block may be too restrictive. Airoldi et al. (2008) proposes an extension of SBMs, called the mixed membership stochastic blockmodels, to allow nodes to belong to multiple groups with varying affiliation probabilities. SBMs have also been extended to accommodate differences in nodal degrees (total number of edges a node has to other nodes) (e.g., Karrer and Newman, 2011), to model network evolution across time (e.g., Xing et al., 2010; Yang et al., 2011; Xu and Hero, 2014; Matias and Miele, 2017) and to model multiple types of relationships among the same set of nodes (e.g., Paul et al., 2016; Barbillon et al., 2017; Paul and Chen, 2020) and among different types of nodes (e.g., Sengupta and Chen, 2015; Huang et al., 2020).

### 2.2. Distance Models

A latent space is a hypothetical multidimensional space that represents the positions of nodes in a lower dimensional space. The position of nodes in the lower dimensional space reflects the geometric rules of the latent distance model. The idea of a latent space for network modeling is first seen in Hoff et al. (2002), where a vector of length *K*, ***u*** is used to represent the position of a node in a *K*-dimensional latent space. Conditional on the latent positions, the probability of two nodes forming an edge is independent of all the other edges in the network:


(3) $$\begin{array}{l} P\left(X|U,Z,\beta \right)=\overset{N}{\underset{a=1}{\prod }}\overset{N}{\underset{b=1,b\neq a}{\prod }}p\left(x_{a,b}|u_{\text{a}},u_{\text{b}},z_{a,b},\beta \right), \end{array}$$

where ***U*** is a *K* × *N* matrix consisting of latent positions, and ***Z***, ***β*** include the covariates and their coefficients.

In this section, I review network modeling with continuous latent variables following the categorization of distance vs. vector models. In a vector model, the vector product between two nodes is included, thus the vector length and the angle between two vectors both drive the interpretation of the latent space. In a distance model, the Euclidean distance between two nodes is included, thus the magnitude of the distance drives the interpretation of the latent space. The smaller the Euclidean distance, the more likely that the two nodes form a connection.

#### 2.2.1. Latent Distance Model

The probability of an edge between two nodes depends on their Euclidean distance given covariates (Hoff et al., 2002):


(4) $$\begin{array}{l} E\left(x_{a,b}|u_{a},u_{b},z_{a,b},\beta \right)=g\left(\phi_{a,b}\right)\\ \phi_{a,b}=\alpha +\beta^{T}z_{a,b}-|u_{a}-u_{b}|, \end{array}$$

where α is the intercept; *g*(·) is the link function. Most commonly, ***X*** is binary; *g*(ϕ_*a,b*_) is the logistic inverse link functions, i.e., $g\left(\phi_{a,b}\right)=\frac{\exp\left(\phi_{a,b}\right)}{1+\exp\left(\phi_{a,b}\right)}$. Other link functions for the latent distance model are available in the latentnet package (Krivitsky and Handcock, 2008).

The latent distance model has been extended to allow built-in clustering, called the latent position cluster model (Handcock et al., 2007), to accommodate nodes' varying degrees of sociability and popularity, called the latent cluster random effects model (Krivitsky et al., 2009), to model multi-layer networks (Gollini and Murphy, 2016; Salter-Townshend and McCormick, 2017; Zhang et al., 2020), and to model network's changes across time (Sarkar and Moore, 2005; Sewell and Chen, 2015). The latent distance model can be fitted using the latentnet package with MCMC algorithms. For large datasets, the latent distance model can be fitted using the lvm4net package with variational inference (Gollini, 2014).

### 2.3. Vector Models

#### 2.3.1. Latent Projection Model

The earliest form of vector network model appears in Hoff et al. (2002), called the latent projection model:


(5) $$\begin{array}{l} \phi_{a,b}=\alpha +\beta^{T}z_{a,b}+\frac{u_{a}^{T}u_{b}}{\left||u_{b}|\right|}. \end{array}$$

In the projection model, the angle between two latent vectors and the length of vector ***u***_*a*_ affect two nodes' probability to connect. When looking at a latent space formed by the latent projection model, one can assess nodes' propensity to form edges by looking at vectors' directions, with opposite directions ($u_{a}^{T}u_{b}<0$) indicating aversion to edge formation, similar directions ($u_{a}^{T}u_{b}>0$) indicating favorable edge formation, and perpendicular relations ($u_{a}^{T}u_{b}=0$) indicating independence. The probability of a connection from node *a* to node *b* is differently modeled than the probability of a connection from node *b* to node *a*.

#### 2.3.2. Bilinear Mixed-Effects Model

Hoff (2005) proposed a generalized bilinear mixed-effects model that can be seen as an extension of the latent projection model with a modified latent effect:


(6) $$\begin{array}{l} \phi_{a,b}=\beta^{T}z_{a,b}+u_{a}^{T}u_{b}+a_{a}+b_{b}+\gamma_{a,b} \end{array}$$

$$\begin{array}{l} \;(a_{a},b_{b})\sim MVN\left(0,\Sigma_{ab}\right),\\ \left(\gamma_{a,b},\gamma_{b,a}\right)\sim MVN\left(0,\Sigma_{\gamma }\right),\\ \;u\sim MVN\left(0,\sigma_{u}^{2}I_{k}\right), \end{array}$$

where *a*_*a*_ is the random sender (initiator of a connection) effect, *b*_*b*_ is the random receiver (receiver of a connection) effect, and **Σ*_γ_*** represents the within dyad (a pair of nodes) dependence. Higher reciprocity means that the receiver of a connection is more likely to reciprocate the friendly gesture by the sender. Across the row of the social network, differences in nodes' sociability are accounted for by *a*_*a*_; and, across columns, differences in nodes' popularity are accounted for by *b*_*b*_. Dependencies occurring in a group of three nodes or more include transitivity, balance and clusterability; they are accounted for by the vector product $u_{a}^{T}u_{b}$. We refer readers to Wasserman and Faust (1994) and Hoff (2008) for further discussions about these dependencies.

#### 2.3.3. Additive and Multiplicative Effects Model

The additive and multiplicative effects (AME) model is proposed in Minhas et al. (2019) and Hoff (2021).


(7) $$\begin{array}{l} \phi_{a,b}=\beta^{T}z_{a,b}+u_{a}^{T}Dv_{b}+a_{a}+b_{b}+\gamma_{a,b} \end{array}$$

$$\begin{array}{l} \;(a_{a},b_{b})\sim MVN\left(0,\Sigma_{ab}\right),\\ \left(\gamma_{a,b},\gamma_{b,a}\right)\sim MVN\left(0,\Sigma_{\gamma }\right),\\ \;(u,v)\sim MVN\left(0,\Sigma_{uv}\right), \end{array}$$

where $u_{a}^{T}Dv_{b}$ is the multiplicative effect modeling dependencies involving triads of nodes. Models with a different multiplicative effect ($u_{a}^{T}Du_{b}$) are sometimes called eigenmodels or latent factor models (see Hoff, 2008; Minhas et al., 2019). Another similar but different multiplicative effect can be seen in Hoff (2021) without the diagonal matrix ***D***, which allows different latent dimensions to exert different effects on the probabilities to connect. Other parameters of the AME model follow those of the bilinear mixed-effects model. The AME model can be fitted using the AMEN package (Hoff et al., 2020).

The bilinear mixed-effects model and AME model are extensions of the latent projection model. According to Hoff (2008), the multiplicative effect in the eigen model, ***u***^*T*^***Du***, generalizes the latent class effect in stochastic blockmodels and the latent distance effect in latent distance models. Sosa and Buitrago (2021) show that the eigen model has better out of sample predictive accuracy for networks with varying properties and structures compared to the latent class and distance models. We refer readers to Smith et al. (2019) and Sosa and Buitrago (2021) for further discussions on vector models vs. distance models.

In addition to latent spaces defined by Euclidean distances and vector products, Schweinberger and Snijders (2003) proposes an ultrametric space, where nodes are assigned to a system of nested groups. A negatively curved hyperbolic space has been proposed by Krioukov et al. (2010). The radius of the hyperbolic space grows exponentially rather than linearly, as in Euclidean distance space. A hyperbolic space is more suitable for networks with tree-like structures (see Krioukov et al., 2010; Smith et al., 2019).

## 3. Introducing Latent Variable Network Modeling to Psychometric Models

Recent research has seen a surge of developments integrating network models with psychometric models such as structural equation models and item response models. In this section, I summarize some of these efforts.

### 3.1. Doubly Latent Space Joint Model

Jin and Jeon (2019) proposes the doubly latent space joint model (DLSJM) that applies the latent space model framework to item responses. The DLSJM separates the information about items and persons in the item response matrix, ***Y*** into two sets of sociomatrices ***V*** = {***V***_1_, ***V***_2_, …***V***_*N*_} and ***U*** = {***U***_1_, ***U***_2_, …***U***_*M*_}, where *N* is the total number of persons, and *M* is the total number of items. I will use subscripts *p* and *j* as person indices and subscripts *a* and *b* as item indices.


(8) $$\begin{array}{l} u_{p,ab}=\{\begin{array}{cc} 1, & \text{if }y_{p,a}=1\&y_{p,b}=1\\ 0, & \text{otherwise},p=1,2,\ldots ,N,a,b=1,2,\ldots ,\\ \; & M,a\neq b \end{array}\\ v_{a,pj}=\{\begin{array}{cc} 1, & \text{if }y_{p,a}=1\&y_{j,a}=1\\ 0, & \text{ otherwise},a=1,2,\ldots ,M,p,j=1,2,\ldots ,\\ \; & N,p\neq j. \end{array} \end{array}$$

This transformation is a projection of the item response matrix, seen as a bipartite network, into sets of relationship sociomatrices that record the similarity of items and the commonality of persons. In network terms, this transformation relies on the *duality* of bipartite networks, the transfer of information between connections within nodes of type *A*, connections within nodes of type *B* and connections between nodes of type *A* and nodes of type *B*.

After transformation, DLSJM models the commonality of persons as a network and estimates latent positions of persons *p* and *j* following the latent distance model:


(9) $$\begin{array}{l} P\left(U,V|Z,\beta ,\theta \right)=\overset{N}{\underset{p=1}{\prod }}P\left(U_{p}|Z,\theta_{p}\right)\overset{M}{\underset{a=1}{\prod }}P\left(V_{a}|Z,\beta_{a}\right)\\ \;=\overset{N}{\underset{p=1}{\prod }}\underset{a\neq b}{\prod }\frac{\exp{\left(\theta_{p}-|f_{a}\left(z\right)-f_{b}\left(z\right)|\right)}^{u_{p,ab}}}{1+\exp\left(\theta_{p}-|f_{a}\left(z\right)-f_{b}\left(z\right)|\right)}\\ \;\overset{M}{\underset{a=1}{\prod }}\underset{p\neq j}{\prod }\frac{\exp{\left(\beta_{a}-|z_{p}-z_{j}|\right)}^{v_{a,pj}}}{1+\exp\left(\beta_{a}-|z_{p}-z_{j}|\right)}\\ \;f_{a}\left(z\right)=\overset{N}{\underset{p=1}{\sum }}\frac{y_{p,a}z_{p}}{\sum_{p=1}^{N}y_{p,a}},\;f_{b}\left(z\right)=\overset{N}{\underset{p=1}{\sum }}\frac{y_{p,b}z_{p}}{\sum_{p=1}^{N}y_{p,b}}, \end{array}$$

where ***z***_***p***_ and ***z***_***j***_ are latent positions for persons *p* and *j*; *f*_*a*_(***z***) and *f*_*b*_(***z***) are functions of D-dimensional latent person positions served as latent positions of items. Latent item positions are defined as averages of positive responses weighted by latent person positions, or alternatively speaking, averages of latent person positions for whom the responses are positive. A hierarchical extension of the DLSJM is proposed by Jin et al. (2018) to accommodate variations across different schools.

Following latent distance model and latent space joint model (Gollini and Murphy, 2016), DLSJM takes on similar model assumptions. In DLSJM, sociomatrices ***V*** and ***U*** are conditionally independent given latent person positions. Furthermore, information collected for person *i* in the form of pairwise similarities of all items are assumed to be conditionally independent to similar information collected for person *j*. Information collected for item *a* in the form of commonalities of all persons are also assumed to be conditionally independent to similar information collected for item *b*.

### 3.2. Latent Space Item Response Model

Jeon et al. (2021) proposes a latent space item response model, which applies a latent distance model with random intercepts to an item response matrix. For a binary item response matrix ***Y***, the expectation of a correct response for person *p* to item *i* is as follows:


(10) $$\begin{array}{l} E\left(y_{p,i}|\alpha_{p},\beta_{i},u_{p},v_{i}\right)=\alpha_{p}+\beta_{i}+\gamma |u_{p}-v_{i}|, \end{array}$$

where $u_{p}\overset{iid}{\sim }N\left(0,{\text{I}}_{D}\right)$, $v_{i}\overset{iid}{\sim }N\left(0,{\text{I}}_{D}\right)$, $\alpha_{p}\overset{iid}{\sim }N\left(0,\sigma^{2}\right)$, $\beta_{i}\overset{iid}{\sim }N\left(0,\tau_{\beta }^{2}\right)$, log $\gamma \sim N(u_{\gamma },\tau_{\gamma }^{2})$, $\sigma^{2}\overset{iid}{\sim }\text{Inv-Gamma}\left(a_{\sigma },b_{\sigma }\right)$, and *D* is the number of dimensions. Following notations in the previous sections, I use ***u***_***p***_ to denote the latent position of person *p* and ***v***_***i***_ to denote the latent position of item *i*. The authors use random effects α_*p*_ and β_*i*_ to account for differences in the overall scores of persons and in the sum scores of items.

Compared with DLSJM, the latent space item response model analyzes item responses instead of first transforming item responses. Compared to Rasch models with random item effects, it adds a Euclidean-distance latent effect to capture deviations from the random intercepts. The added Euclidean-distance latent effect also allows visualizations of the item and person nodes in a hypothetical latent space. As a network model, latent space item response model can be seen as a bipartite latent space model, first developed in the latentnet package (Krivitsky and Handcock, 2008). Unlike the bipartite latent space model, the latent space item response model adds a γ coefficient in front of the Euclidean-distance latent effect, allowing varying degrees of contribution by the Euclidean-distance effect to the probability of a connection.

Latent space item response model can be fitted using the latentnet package with the assumption of γ = −1. For large-scale datasets, a variational algorithm for bipartite latent space model is proposed by Wang et al. (2021), which can be applied using the jlsm package (Wang, 2021). The bipartite latent space model has also been extended to model networks across multiple time points by Sarkar and Moore (2005) and Friel et al. (2016).

### 3.3. Social Network Structural Equation Model

Liu et al. (2018) proposes social network structural equation model that integrates latent distance model with structural equation model. Different from the previous two methods, the social network structural equation model analyzes both a network dataset and an item response matrix with the goal of estimating their dependence or association. Following previous notations, I will use ***X*** to denote the social network, ***Y*** to denote the item response matrix and ***Z*** to denote covariates. The model is estimated in two separate steps. First, item responses are fitted to a confirmatory factor model (measurement model):


(11) $$\begin{array}{l} y_{p}=\Lambda \eta_{p}+\epsilon_{p},p=1,2,\ldots ,N, \end{array}$$

where ***y***_*p*_ is a vector of length *M* representing person *p*'s scores on *M* items (indicators); ***η***_*p*_ represents a vector of *D* latent variables, ***η***_*p*_ ~ *MVN*(0, **Φ**); **Λ** is the *M* × *D* factor loading matrix; and **ϵ**_*p*_ is the unexplained residual, **ϵ**_*p*_ ~ *MVN*(0, **Ψ**). In the second step, the factor scores from the first step are fitted as covariates of the social network model (structural model):


(12) $$\begin{array}{l} E\left(x_{a,b}|\alpha ,\beta ,\gamma \right)=\alpha +\beta^{T}h_{a,b}+\gamma \sqrt{{\left(\eta_{a}-\eta_{b}\right)}^{T}\Phi^{-1}\left(\eta_{a}-\eta_{b}\right)}, \end{array}$$

where α is the intercept; ***h***_*a,b*_ is the manifest nodal covariate between nodes *a* and *b*^2^; and ***η***_*a*_ and ***η***_*b*_ are the factor scores obtained from the measurement model. Coefficients ***β*** and ***γ*** estimate the effects of the manifest covariates and the Mahalanobis distances of the latent factors, respectively. In the structural model, the friendship connection is explained by manifest covariates as well as factor scores extracted from the measurement model. The measurement model can be fitted using the lavvan package (Rosseel, 2012), and the structural model can be fitted as a logistic regression.

Extensions of the social network structural equation model are proposed as social network mediation analysis, where the latent position is first extracted from the social network following latent distance model (Liu et al., 2021) and eigenmodel (Che et al., 2021), and subsequently fitted as a mediator in a regression analysis. The latent positions can be estimated using the latentnet package (Krivitsky and Handcock, 2008), the Eigenmodel package (Hoff, 2012), or the AMEN package (Hoff, 2015). Social networks have been fitted as regression mediators in the hierarchical network model (HNM) for mediation, proposed by Sweet (2019). In HNM for mediation, a network statistic is used to summarize the entire network and then fitted as the mediator in a regression model. Different from social network mediation analysis, the network model and the mediation model in HNM for mediation are simultaneously estimated instead of in two steps.

### 3.4. Joint Latent Space Model

Wang et al. (2021) proposes a joint latent space model (JLSM) to jointly analyze social network and item responses. Using a joint modeling approach, the authors model social network and item responses stemming from a shared data generation process. I use ***u***_***a***_ to denote latent position of person *a* and ***v***_***i***_ to denote latent position of attribute *i*.


(13) $$\begin{array}{l} p\left(X,Y|U,V,\alpha_{0},\alpha_{1}\right)=\overset{N}{\underset{a=1}{\prod }}\overset{N}{\underset{b=1,b\neq a}{\prod }}p\left(x_{a,b}|\alpha_{0},u_{a},u_{b}\right)\overset{N}{\underset{p=1}{\prod }}\\ \;\overset{M}{\underset{i=1}{\prod }}p\left(y_{p,i}|\alpha_{1},u_{p},v_{i}\right),\\ \;\overset{N}{\underset{a=1}{\prod }}\overset{N}{\underset{b=1,b\neq a}{\prod }}\frac{\exp{\left(\alpha_{0}-|u_{a}-u_{b}{|}^{2}\right)}^{x_{a,b}}}{1+\exp\left(\alpha_{0}-|u_{a}-u_{b}{|}^{2}\right)}\\ \;\overset{N}{\underset{p=1}{\prod }}\overset{M}{\underset{i=1}{\prod }}\frac{\exp{\left(\alpha_{1}-|u_{p}-v_{i}{|}^{2}\right)}^{y_{p,i}}}{1+\exp\left(\alpha_{1}-|u_{p}-v_{i}{|}^{2}\right)}, \end{array}$$

where $u_{a}\overset{iid}{\sim }MVN\left(0,\lambda_{0}^{2}{\text{I}}_{D}\right)$, $v_{i}\overset{iid}{\sim }MVN\left(0,\lambda_{1}^{2}{\text{I}}_{D}\right)$, and $\alpha_{0},\alpha_{1},\lambda_{0}^{2},\lambda_{1}^{2}$ are unknown parameters. To model the connection between persons *a* and *b*, the authors use the (squared) Euclidean distance^3^ between latent positions ***u***_***a***_ and ***u***_***b***_. To model whether person *p* possesses attribute *i*, the authors use the Euclidean distance between latent positions ***u***_***p***_ and ***v***_***i***_. The dependence between social network and item responses is modeled by the shared latent variable ***U***.

JLSM^4^ extends the latent space joint model proposed by Gollini and Murphy (2016), and both use a shared latent variable to model the dependence between different networks. The JLSM can be applied using the jlsm package (Wang, 2021).

## 4. Network Psychometrics

Although not a framework for modeling networks, *network psychometrics* has gain wide-spread popularity in various disciplines of psychology (e.g., Cramer et al., 2012; Fried et al., 2015; McNally et al., 2015; van Borkulo et al., 2015; Dalege et al., 2016; Isvoranu et al., 2016; Kossakowski et al., 2016) with a mission to contend the common-cause interpretation of latent variables with a web (network) of connected variables. Whether the contention is fair or a matter of incomplete perception (see Fried and Cramer, 2017; Bringmann and Eronen, 2018), it has paved the way for the introduction of network modeling to psychology. For this reason, we provide a summary of current developments under network psychometrics, and we offer a few key distinctions of this framework from the current discussion.

Graphical models are proposed under network psychometrics to analyze multivariate datasets including item responses. Each variable is conceptualized as a node in the network, and the presence of conditional dependence between variables is constructed as an edge between two nodes. In graphical models, the network is no longer the observed (social) network, but constructed based on correlations between observed variables. The gaussian graphical model (GGM) constructs edges between variables based on their partial correlations. Suppose we observe *P* multivariate normal variables, *y*_*j*_, *j* = 1, 2, …, *P*:


(14) $$\begin{array}{l} \text{Cor}\left(y_{j},y_{k}|y^{-\left(j,k\right)}\right)=w_{jk}=w_{kj}, \end{array}$$

where *w*_*jk*_ is the weight of the edge between nodes *j* and *k*. Compared with latent variable models such as structural equation model, GGM considers correlations between observed variables as the data generating process instead of outcomes of shared latent variables.

In Marsman et al. (2018), it is shown that the Ising model is statistically equivalent to the item response model such that we can expect the same model fit when both are applied to the same data. By proving this equivalence, Marsman et al. (2018) provides an alternative framework for understanding abstract psychological phenomena such as depression. The co-occurrence of disorder symptoms can be hypothesized as stemming from correlated observed variables instead of a shared latent variable. This alternative view has broad applications in various sub-fields of psychology.

In latent network modeling, Epskamp et al. (2017) combines graphical models with latent variable models by allowing latent variables (or their residuals, seen in residual network modeling) to be modeled as a correlated network. Epskamp (2020) extends GGM to model observations across time, called graphical vector autoregressive model. Yang et al. (2014) and Chen et al. (2015) propose mixed graphical models to accommodate situations, where distributions of variables, conditional on other variables, have different exponential family forms. I refer readers to Epskamp et al. (2017), Epskamp (2020), and Altenbuchinger et al. (2020) for comprehensive discussions of graphical models under network psychometrics. These graphical models can be implemented using the qgraph package (Epskamp et al., 2012); and a review of related statistical software can be seen in Haslbeck and Waldorp (2015).

Compared with network models introduced in the previous sections, graphical models under network psychometrics possess a few key distinctions. First, these models are not meant for analyzing networks, instead they create a network representation of variables based on correlations. Second, as a psychological framework, network psychometrics presents an alternative way to conceptualize psychological phenomena, which is not the case for network modeling. It argues for a network of correlated observed variables as the true data generating process instead of shared latent variables. Furthermore, network models are distinct from other analytic methods because networks are distinct from other types of data including multivariate datasets. The assumption of independence does not apply in a network as it does in a multivariate dataset because the association between persons is part of the modeling interest. In fact, dependencies in a network are often complex and are not yet well-defined. For these reasons, network modeling, as a discipline, is often not covered in quantitative courses, and multivariate models are not applicable for network datasets.

## 5. Summary and Directions for Future Research

**Figure 1:**
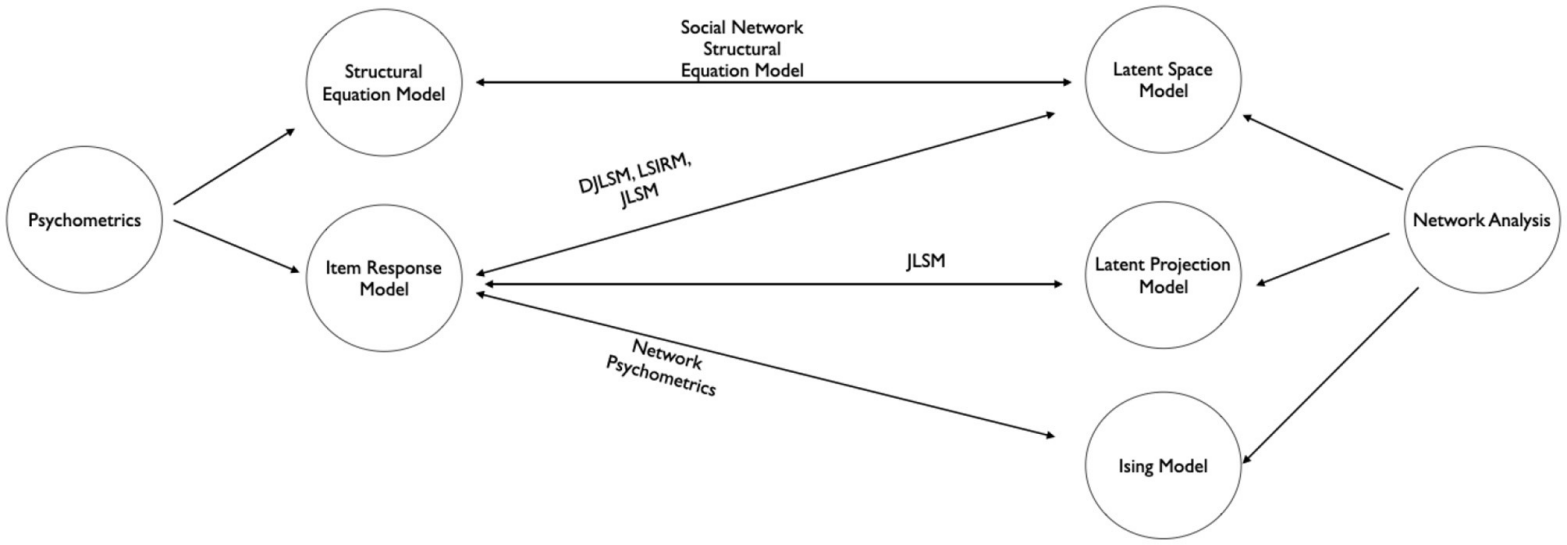
Diagram summarizing the relationship between psychometric models and network models^5^. Double-headed arrows indicate integrations of two frameworks with the resulting framework at the center.

Studies on network modeling and psychometrics may be divided into many lines of research, such as dynamic vs. static modeling and Bayesian vs. frequentist inference. In this paper, I focus on the integration of latent variable network modeling with psychometric models. When analyzing a network, latent variables are used to model network dependencies (e.g., associations between a pair of nodes or a group of nodes) and to construct low-dimensional geometric spaces, which are useful for visualization. In psychometric models, latent variables can be used to simplify model specification and provide statistical representation of abstract latent constructs. From these, I outline recent efforts integrating latent variable network models with psychometric models. To conclude, I provide a diagram (see Figure 1) outlining relationships of methods discussed in sections 2, 3, and 4. Psychometric models and network models are in the left and the right of the diagram. Presence of a link represents the integration of the two methods that motivate the development of the new framework at the center.

Despite recent efforts integrating latent variable network models with psychometric models, there remain several challenges. While viewing item response data as a bipartite network opens doors for new possibilities, further research is needed to understand the implications of this equivalence. In a network, the assumption of independence between persons is violated as dependencies between them are the drivers of a connected network, and the goal of network modeling is to analyze and understand such dependencies. Meanwhile, the independence assumption is traditionally applied in an item response matrix, which makes applying network modeling to item responses problematic. This is not necessarily the case though. If respondents of a survey come from the same school or reside in the same geographic region, it is reasonable to assume dependence between them given the nonzero likelihood of their prior engagement. If we assume dependence between persons in an item response matrix, what are its implications?

To fully take into account potential dependence between respondents of an item response matrix, it is preferential to have observations of respondents' relationships in addition to their attributes. To model the dependence between networks and individual attributes, we can incorporate individual attributes as nodal or edge covariates in network modeling, e.g., in stochastic blockmodels (Sweet, 2015; Mele et al., 2019) and in latent space models (Hoff et al., 2002; Krivitsky and Handcock, 2008; Austin et al., 2013; Fosdick and Hoff, 2015). Alternatively, we can apply social influence models and regard individual attributes as the dependent variable and estimate the effects of the social network on attributes. (Dorans and Drasgow, 1978; Robins et al., 2001; Leenders, 2002; Frank et al., 2004; Fujimoto et al., 2013; Sweet and Adhikari, 2020). The situation is more complicated when individual attributes are multivariate or multidimensional such as the item responses. The social network structural equation model regards item responses as the dependent variable in the first step, and then models the social network as the dependent variable in the second step. The joint latent space approach simultaneously models the social network and item responses as the dependent variables. Both pose as interesting directions of research, but improvements are also possible.

Latent variable network modeling has the potential to be further integrated in educational and psychological research. Through this paper, we hope to inspire readers to begin incorporating some of these methods to their current analysis plans and develop new methods filling in existing research gaps and answering new research questions. Despite the novelty of these models, we have included information about the R packages available for network models as well as the integrative methods in order to facilitate further independent learning.

## Author Contributions

The author confirms being the sole contributor of this work and has approved it for publication.

## Conflict of Interest

The author declares that the research was conducted in the absence of any commercial or financial relationships that could be construed as a potential conflict of interest.

## Publisher's Note

All claims expressed in this article are solely those of the authors and do not necessarily represent those of their affiliated organizations, or those of the publisher, the editors and the reviewers. Any product that may be evaluated in this article, or claim that may be made by its manufacturer, is not guaranteed or endorsed by the publisher.

## Footnotes

^1^Ties from a node to itself are rarely considered. Diagonal entries can be written as 0 though they are omitted from the analysis.

^2^Elements of ***h***_*ab*_ are transformation of the nodal covariates.

^3^The squared Euclidean distances are shown to result in similar latent positions as Euclidean distances while maintaining computational efficiency by Gollini and Murphy (2016).

^4^An earlier version of this model can be seen in Wang (2019), where an ensemble of item response matrices was modeled through a shared attribute similarity matrix, and the vector-product latent effect was used instead of the Euclidean distance in modeling the item responses.

^5^JLSM: A earlier version of JLSM contains the vector product latent effect (Wang, 2019).
